# Stress-Induced Tradeoffs in a Free-Living Lizard across a Variable Landscape: Consequences for Individuals and Populations

**DOI:** 10.1371/journal.pone.0049895

**Published:** 2012-11-20

**Authors:** LeiLani D. Lucas, Susannah S. French

**Affiliations:** Department of Biology, Utah State University, Logan, Utah, United States of America; CNRS, Université de Bourgogne, France

## Abstract

Current life history theory suggests that the allocation of energetic resources between competing physiological needs should be dictated by an individual’s longevity and pace of life. One key physiological pathway likely to contribute to the partitioning of resources is the vertebrate stress response. By increasing circulating glucocorticoids the stress response can exert a suite of physiological effects, such as altering immune function. We investigated the effects of stress physiology on individual immunity, reproduction and oxidative stress, across an urban landscape. We sampled populations in and around St. George, Utah, examining corticosterone in response to restraint stress, two innate immune measures, reproductive output, and the presence of both reactive oxygen metabolites and antioxidant binding capacity, in populations of common side-blotched lizards (*Uta stansburiana*) experiencing variable levels of environmental stress. Additionally, using capture-mark-recapture techniques, we examined the relationships between these physiological parameters and population-level differences. Our results reveal elevated physiological stress corresponds with suppressed immunity and increased oxidative stress. Interestingly, urban populations experiencing the most physiological stress also exhibited greater reproductive output and decreased survival relative to rural populations experiencing less physiological stress, demonstrating a tradeoff between reproduction and life maintenance processes. Our results suggest that environmental stress may augment life history strategy in this fast-paced species, and that shifts in life history strategy can in turn affect the population at large. Finally, the urban environment poses definite challenges for organisms, and while it appears that side-blotched lizards are adjusting physiologically, it is unknown what fitness costs these physiological adjustments accrue.

## Introduction

A major challenge in biology is the understanding of how organisms distribute limited resources among physiological processes. Life history theory predicts that this partitioning should reflect longevity and an organism’s ‘pace of life’, where investment in current reproduction occurs at the expense of survival and future reproductive opportunities [1]–[5]. While studies have demonstrated the presence of such trade-offs [6], it is not clear what regulatory processes underlie these tradeoffs, and how life history decisions translate to larger scales whereby they may influence populations [7].

Reproduction is an energetically costly, but essential component of biological fitness. Reproductive processes limit available resources and thereby influence their allocation among competing mechanisms necessary for an organism’s survival and maintenance [4], [8]–[10]. Current studies provide support for trade-offs between self-maintenance processes and reproduction [11]–[13]. Immune function is a key aspect of individual self-maintenance, because it is necessary for effective response to disease and parasites, and therefore is vital for survival [14]–[15]. However, due to the high energetic cost of coping with infections, immunity can limit resource availability for other processes, including reproduction, thereby potentially decreasing an individual’s fitness [16]. The stress response and corresponding glucocorticoid (GC) release are known to affect virtually all aspects of immune function [17]–[18] critical to self-maintenance. Additionally, increased physiological stress is also related to the overproduction of reactive oxygen metabolites, and without sufficient ability to clear those metabolites, can have direct deleterious effects on tissues and ultimately survival [19]–[20].

Although mechanisms responsible for mediating changes in energetic investment among competing physiological systems are still unclear, stress response likely contributes to the reallocation of resources in vertebrates. Activation of the stress response and GC hormones (adrenal steroids elevated in response to stress) serves a major role in the mobilization and reallocation of resources among physiological systems during stressful events [21]–[24]. Research has demonstrated that stress-induced GC changes may contribute to alterations in resource investment between competing processes [25]–[26]. While some of these relationships have been demonstrated under controlled experimental conditions, they are more complicated under natural conditions, fluctuating in response to a variety of factors, including seasonal energy availability [27]–[30] and reproductive state [28], [31]–[36].

Selective pressures tend to move an animal’s life history toward maximal ‘achievable’ fitness, which may necessitate a reduced lifespan. For example, downstream depression of immunity by GCs may function as a tradeoff to preserve resources for reproduction, ultimately increasing fitness at the expense of individual survival [37]. Indeed, prior work suggests that among free-living animals, the effect of stress on immune function interacts with both energetic and reproductive states, often resulting in divergent outcomes [38]–[41], and GCs may mediate these fluctuations [42]–[43].

Because any factor that affects individual reproduction and survival has the potential to affect population growth, physiological shifts in life history strategy could influence demographic fitness components. For example, if GC hormones induce immunosuppression to preserve resources for reproduction [37], and the costs of mounting an immune response may lead to decreased survival [44], then elevated physiological stress could result in decreased survival across a population. Alternatively, stress responses may instead act to suppress immediate reproductive investment to promote survival. Because reproduction requires endocrine changes that regulate a suite of costly physiological, metabolic, and behavioral changes [8]–[9] it is very energetically costly and thus a likely target for resource conservation during stress.

In the current study, we measured differences in immunity, reproduction, and oxidative stress across four populations of common side-blotched lizard (*Uta stansburiana*), an abundant and territorial species well-suited for repeated sampling. Because population level differences in stress physiology have been observed across populations [45], we sampled populations experiencing varying levels of environmental stress across an urban landscape within the greater St. George area in Utah. We used changes in circulating levels of the GC hormone corticosterone (CORT) as a metric for stress responsiveness, and related physiological differences to population-level changes in survival using Capture-Mark-Recapture (CMR) techniques (ideal when detectability is imperfect). We hypothesized that populations experiencing greater physiological stress would exhibit decreased immunity relative to those experiencing less physiological stress. Specifically, we predicted that increased CORT responses should correspond with decreased immune measures in populations experiencing greater physiological stress, which should also correspond with increased reproductive output and a decrease in population-level survival in this short-lived species. Although increasingly more studies have begun to examine urban stress physiology in free-living populations (reviewed in [46]), results regarding the effect of urban ecology on the stress response are currently mixed, and it is therefore apparent that more work is necessary to elucidate the direction of these effects and how they impact other physiological processes.

Because *U. stansburiana* exhibits a short lifespan with high replacement rates [47], from life history theory we expected elevated physiological stress to correspond with increased reproductive output and decreased population-level survival. Such results would suggest that due to their life history, *U. stansburiana* reallocates energy toward immediate reproductive success instead of self-maintenance processes (i.e., immune function) when experiencing increased stress.

Any factor capable of altering individual reproduction and survival may contribute to population-level changes. Although many studies have investigated the effects of hormones on reproduction [48]–[50] or survival [50]–[51] in wild populations, few have explored the concomitant interactions of physiology, reproduction and survival [52]. Understanding interactions between GC response, immunocompetence, and reproduction will help elucidate potential fitness and population-level consequences of stress.

## Results

### Factors Contributing to Bactericidal Ability in Common Side-blotched Lizards

We used a bactericidal assay to detect an individual’s ability to kill *Escherichia coli* bacteria, a functionally relevant and complement-dependent measure of immunity. This technique was chosen because unlike other immune measures, such as total hemolytic complement activity, the killing of *E. coli* also relies on the presence of natural antibodies and phagocytes, giving a more integrative measure of immunity while also providing an indication of complement activity [53].

We used General Linear Models (GLM) to determine the relationship between individual-level factors (i.e., CORT, sex, month, site) and bactericidal ability. We used a completely random 3-factor mixed model structure and included higher-order interactions. Site and individual sex served as fixed-factors, while CORT response (the change in circulating CORT between baseline and post-stress levels) data were normalized and included as a covariate. Because no differences in baseline CORT concentration were detected across populations, only CORT response was included in the analysis. Because individuals were sampled across three months, and month is correlated with female reproductive stage and is known to correspond with male breeding condition, month of sampling was also included in the GLM analysis as a second level temporal effect and an indirect measure of reproductive state. Because month was included in the model and many individuals were resampled across months, repeated measures analysis was also included using individual as a random effect.

**Figure 1:**
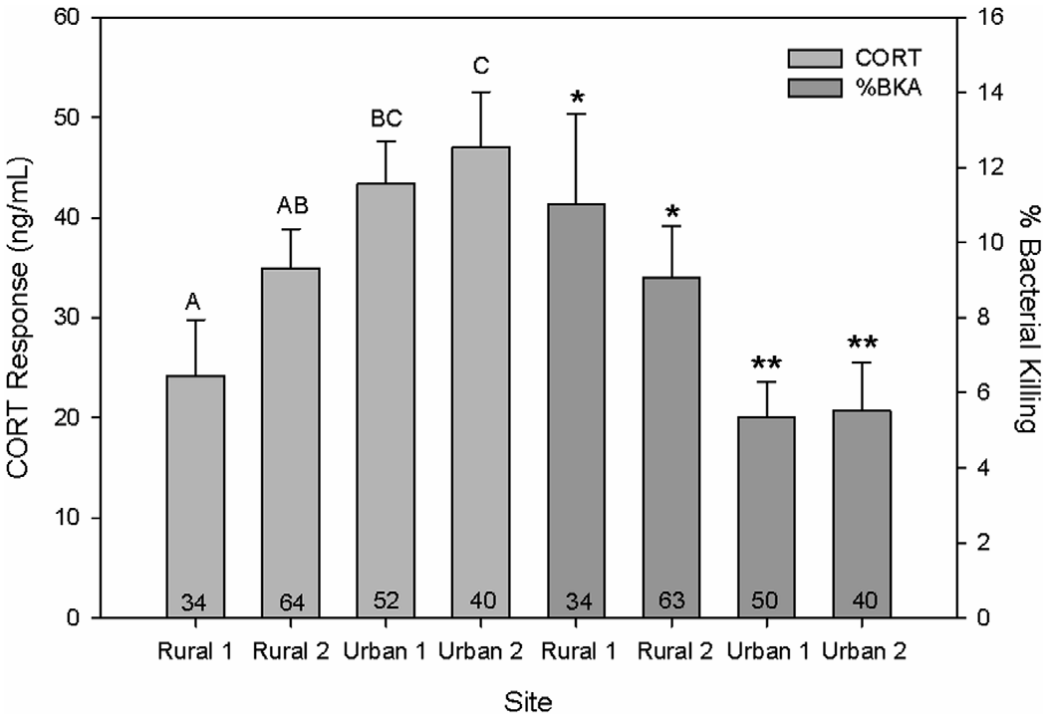
The relationship between CORT and bactericidal ability across sites. A robust negative relationship in which increased CORT response corresponds with decreased ability to kill *E. coli*bacteria was detected in common side-blotched lizards (*P*<0.0001). Individuals sampled from sites within St. George, Utah, exhibited significant decreases in bacterial killing ability compared to those sampled from sites outside St. George (*P*<0.002). Values are mean ±1SE; different letters and symbols denote statistically significant differences. Numbers in bars indicate sample sizes.

**Figure 2:**
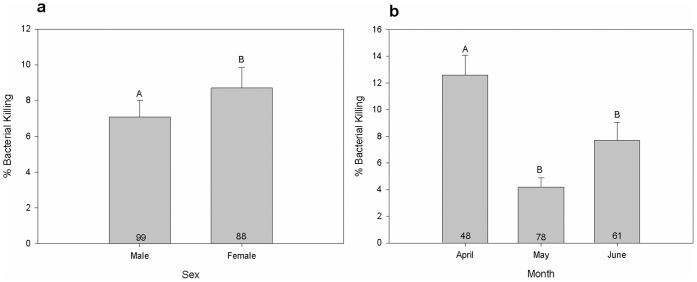
Differences in bactericidal ability between sexes and across months. **a** Males exhibit significantly lower bacterial killing ability relative to females (*P* = 0.02). **b** Significant decreases in bactericidal ability are observed in May and June (months corresponding with increased reproductive investment) relative to April (*P*<0.0001). Numbers in bars indicate sample sizes.

In the most general model that included all additive and interactive terms, fixed effects tests revealed that all first order factors (CORT reactivity, sex, month, and site) demonstrated statistically significant relationships with an individual’s ability to kill *E. coli* bacteria (*P*<0.05), whereas all higher-order interactions did not (*P*>0.05). The magnitude of an individual’s CORT response to restraint stress exhibited a robust negative relationship with bactericidal ability (*F_1,113_* = 17.6, *P*<0.0001), with greater CORT responses corresponding to decreased bactericidal ability (Fig. 1). A significant effect of sex was also detected (*F_1,107_* = 5.94, *P* = 0.02), with females showing slightly greater bactericidal ability relative to males (Fig. 2a). Month of sampling was also strongly related to an individual’s ability to kill *E. coli* bacteria (*F_2,100_* = 18.9, *P*<0.0001), with decreased killing ability observed in May and June (months coinciding with peak reproductive periods) relative to April (a period in which reproductive investment is typically low, Fig. 2b). Finally, comparisons across the four sites sampled showed statistically significant differences in bactericidal ability among sites (*F_2,94_* = 7.41, *P* = 0.002). Post-hoc analysis revealed that the four sites sampled fell into two separate groups, in which both sites sampled within St. George (i.e., urban areas) exhibited decreased bactericidal ability relative to those sampled outside of St. George (i.e., rural areas; Fig. 1).

### Wound Healing

Wound healing involves multiple immune phases (inflammatory response and cellular proliferation and mobilization) and therefore provides an integrative measure of innate immunity with a clear endpoint [54]. Individuals were sampled from subpopulations from two of our four primary sites.

**Figure 3:**
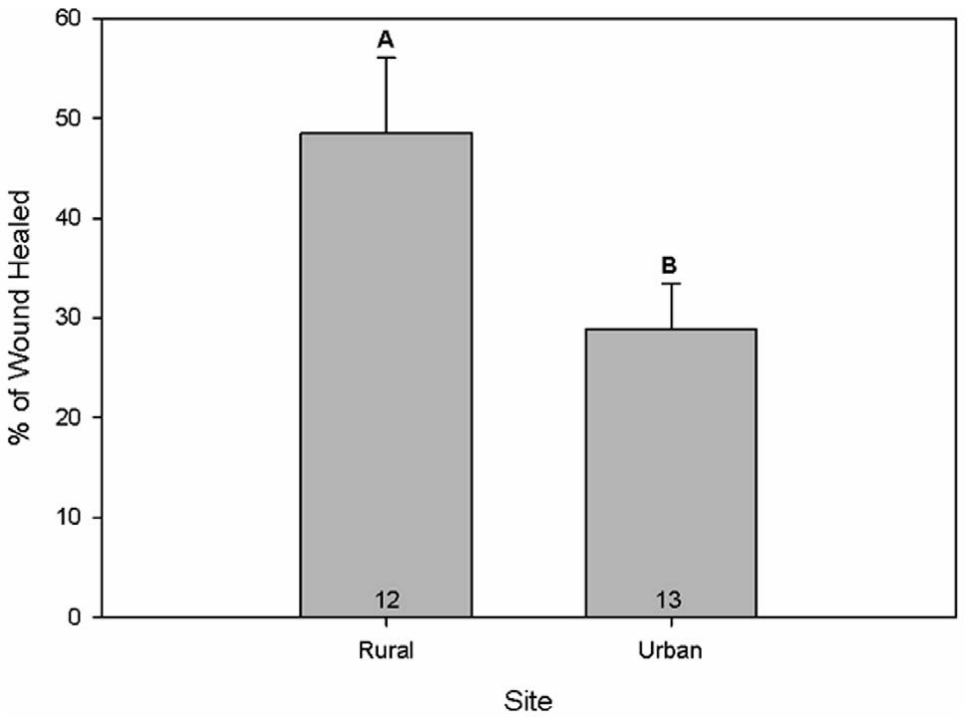
Wound healing between sites. Percent of a cutaneous wound healed by common side-blotched lizards from urban and rural sites after 10 days. Rural lizards demonstrated greater wound healing compared to urban lizards (*P* = 0.05). Numbers in bars indicate sample sizes.

Because we were unable to detect sex-specific differences in wound healing (*t* = 0.35, *df* = 1, *P* = 0.73), males and females were pooled together for statistical analysis. We performed a *t* test to detect differences in the percent wound healing 10 days after wounding with a cutaneous biopsy punch. As with the bactericidal data, individuals sampled within St. George demonstrated statistically significant decreases in their ability to heal a cutaneous wound compared to individuals sampled outside of St. George (*t* = −2.03, *df* = 1, *P* = 0.05, Fig. 3).

### Oxidative Stress

The relationship between circulating CORT and oxidative stress was analyzed using blood samples collected in May 2010 from all four of our study populations. Because an accurate measure of oxidative stress requires knowledge of both the presence of reactive oxygen metabolites (ROMs) and the capacity to bind to and clear those metabolites, we quantified both the presence of ROMs and total non-enzymatic antioxidant capacity (OXY). An index of plasma oxidative status (OI), measured as the difference between the ROMs and the OXY standardized values [55], was calculated to provide an integrative measure of oxidative stress. The relationship between CORT and oxidative stress was measured using a standard least squares regression analysis between OI and CORT response. To determine which factor(s) contributed to the overall effect of CORT on OI, separate standard least squares regressions were also performed for the relationships between CORT and both ROMs and OXY. Site differences in OI were tested using a one-way ANOVA.

**Figure 4:**
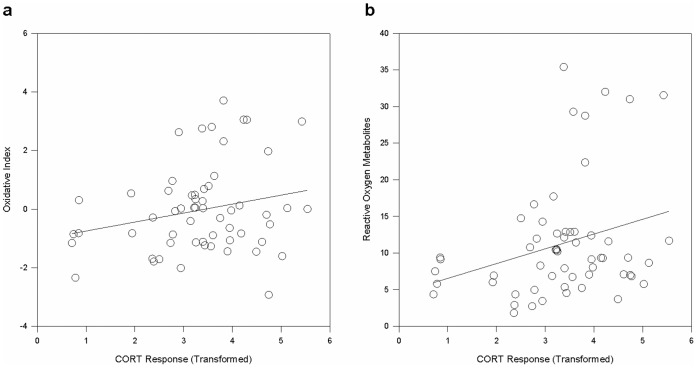
The relationship between CORT response and oxidative stress. **a** A significant positive relationship between an individual’s Oxidative Index (an integrative index of plasma oxidative status) and CORT response was observed in common side-blotched lizards, indicating that increased stress reactivity corresponds with elevated oxidative stress (*P* = 0.03, *n* = 63). **b** The presence of reactive oxygen metabolites (measured as mM of H_2_O_2_) exhibited a significant positive relationship with CORT response (*P* = 0.02, *n* = 63), suggesting that stress-induced increases in the presence of reactive oxygen metabolites may drive individual variation in oxidative stress in this species.

Our results revealed a positive relationship in which elevated endogenous CORT concentration corresponds with an increased OI (*F_1,56_* = 4.99, *P* = 0.03, Fig. 4a), suggesting that individuals with greater CORT reactivity experience greater levels of oxidative stress. This relationship appears to be driven by an individual’s level of ROMs, which were shown to increase as baseline CORT increased (*F_1,56_ = *6.03, *P* = 0.02, Fig. 4b), whereas a significant relationship between an individual’s OXY and baseline CORT was not detected (*F_1,56_ = *0.56, *P*>0.05). Statistically significant differences in OI across sites were not detected (*F_3,57_* = 0.89, *P*>0.05).

### Reproductive Output

Separate one-way ANOVAs were performed to test for differences in clutch size and egg diameter across sites. Standard least squares regression were also performed to analyze the relationship between CORT reactivity and both clutch and egg/follicle size.

**Figure 5:**
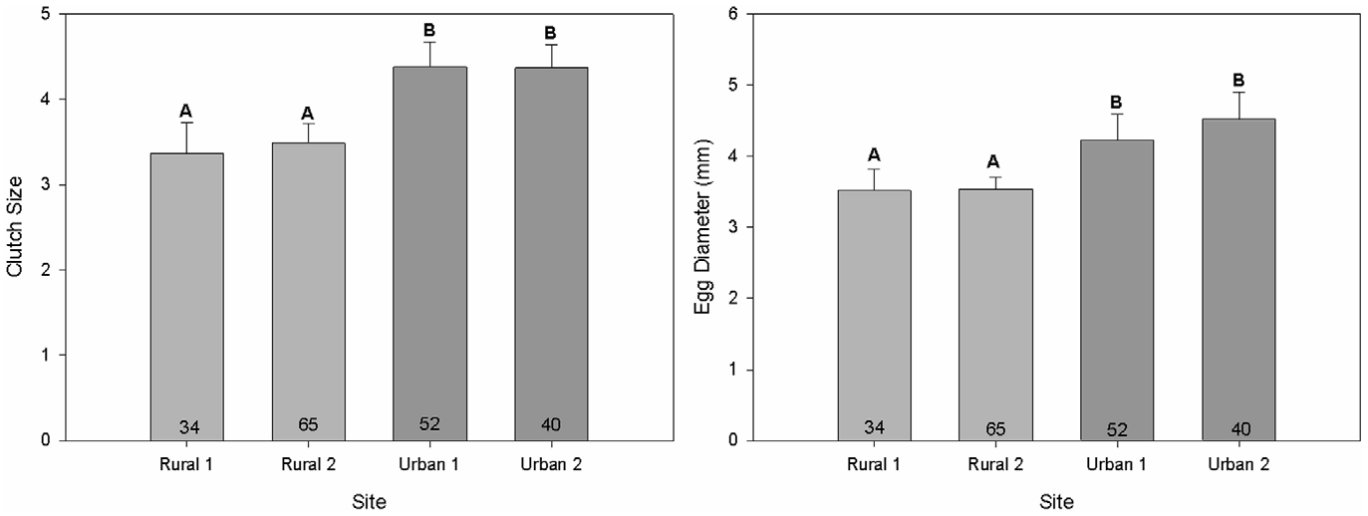
Reproductive output across sites. **a** Common side-blotched lizards sampled within the city of St. George, Utah, were found to produce larger clutches relative to those sampled outside of St. George (*P* = 0.02*)*. **b** Urban individuals produced eggs of slightly greater diameter compared to rural counterparts (*P* = 0.05). Values are mean ±1SE; different letters denote statistically significant differences.

Significant differences in both clutch size (*F_3,78_ = *3.63, *P* = 0.02) and egg diameter (*F_3,77_ = *2.70, *P* = 0.05) were detected across sites. Post-hoc comparisons for both tests revealed a similar pattern as that detected in the bactericidal GLM analysis, with individuals sampled within St. George exhibiting statistically significant differences in clutch size (Fig. 5a) and egg diameter (Fig. 5b) compared to those sampled outside of St. George. Specifically, significantly larger clutches and eggs were observed among individuals from urban sites relative to those from rural sites. Additionally, egg diameter (*F_1,58_ = *6.62, *P* = 0.01), but not clutch size (*F_1,59_ = *0.04, *P* = 0.84) was shown to exhibit a statistically significant positive relationship with stress-induced CORT response.

### Population Analyses

Survival was estimated for each site across the three-month sampling period (April-June 2010) using robust design CMR statistical estimation. Because CMR design allows for survival estimations based on dynamic physiological states and other phenotypic characteristics, we were able to include CORT reactivity, bactericidal ability and sex as covariates to determine how these parameters were driving differences in survival among these populations. Nine candidate models incorporating various combinations of our covariates were generated based on biological assumptions of interest. Models were compared against one another, including a null model that did not include covariates and assumed no differences in survival between populations.

Because only two of our candidate models revealed confidence intervals that did not overlap zero, and confidence intervals overlapping zero suggest that those survival estimates most likely are not significant and survival estimates generated by these models may not be accurate, we will focus on our two best candidate models. For additional information on model selection and our candidate models, please see Table S1 for a description of candidate models and a summary of results for each candidate model.

The best model tested included only CORT reactivity as a covariate. Further, given the ΔAIC_c_ of this model and all other candidate models, and a comparison of AIC_c_ weights, our data strongly suggest that CORT response is an important driving factor in the variation in survival observed in our populations of *U. stansburiana*. The second best model is one that included only bacterial killing ability as a covariate. Although we detected statistically significant differences between these two models, as indicated by the ΔAIC_c_, both models attained similar levels of survival across sites (Table 1). Thus, while our data suggest that CORT reactivity exerts the greatest influence on survival, bactericidal ability may also be a significant factor. The inaccurate estimates provided by a candidate model that included an additive effect of CORT reactivity and bacterial killing ability may result from not having enough statistical power (i.e., limited sample size) to include two covariates into the model.

**Table 1:** Beta parameters and survival estimates of top candidate models.

Model	β_Intercept_	β_covariate_	Site	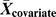	Survival(φ)
φ(CORT)	2.1884147	−0.0629652	Rural 1	24.977	0.65
			Rural 2	38.128	0.45
			Urban 1	43.374	0.37
			Urban 2	47.017	0.32
φ(BKA)	−1.4763462	0.1510933	Rural 1	11.020	0.55
			Rural 2	9.061	0.47
			Urban 1	5.354	0.34
			Urban 2	5.514	0.34

The top model, φ(CORT), shows a negative relationship between CORT response and survival, while the second best model, φ(BKA), shows a positive relationship between bactericidal ability and survival. Population means for covariates, 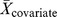
_,_ reveal that urban populations exhibit heighted CORT response and decreased bacterial killing ability concomitant with decreased survival rates relative to rural populations.

Survival estimates reveal similar differences among populations as those observed in the physiological data previously described. Beta values for the model including CORT reactivity as a covariate indicate a negative relationship between CORT and survival, in which increased CORT response corresponds with decreased survival. The model including bactericidal ability as a covariate indicates a positive relationship where increased bacterial killing ability is related to increased survival. Additionally, as with the physiological data, the four sites sampled seem to fall into two separate groups, in which the two populations located within St. George (i.e., urban populations) exhibit similar levels of decreased survival relative to those populations located outside of St. George (i.e., rural populations). While there appears to be more variability in survival between the rural sites than the urban sites, our models suggest that urban sites exhibit higher investment in reproduction and lower investment in immunity, which corresponds with decreased survival.

## Discussion

Observed site differences in physiological responses to stress were pervasive and consistent throughout our study. Individuals from sites located within the city exhibited decreases in immunity while simultaneously showing increases in reproductive output relative to individuals located outside of St. George. Increased stress reactivity was also observed in urban versus rural lizards, further suggesting that the stress mechanisms may play a role in mediating tradeoffs between immunity and reproduction. Survival estimates indicating markedly lower survival in urban populations relative to rural populations when expressed as a function of CORT and bactericidal ability further suggest the relevance of the relationship between immunity and stress. While it is possible that survival estimates may be influenced by differences in movement patterns among populations, this seems unlikely given the territoriality and relatively small home range sizes of this species [56]–[57].

Our population data also demonstrates that survival exhibits a negative relationship between CORT reactivity while concomitantly exhibiting a positive relationship between bactericidal ability in this species. Thus, our data supports the hypothesis that a shift in investment in self-maintenance may be occurring across populations that are exposed to varying levels of environmental perturbation. Other factors, however, may also contribute to the observed population differences. For example, environmental pressures may have selected for stress-resistant genotypes in urban populations to cope with the persistent perturbations experienced in urban environments. Alternatively, urban individuals may be exposed to environmental toxins that result in a priming of the stress response. While, additional work is necessary to rigorously test observed differences between urban and rural populations, currently our results provide support for the existence of tradeoffs between self-maintenance processes and reproductive investment, the mediation of which is related to stress response. These tradeoffs also appear to translate into population-level changes by affecting fitness components such as reproductive output and survival. This study provides one of the most comprehensive investigations of interactions between immunity, reproductive output, survival and stress physiology in natural populations to date.

### Physiological Tradeoffs

Specifically, decreased bactericidal ability corresponds with peak periods of reproduction, suggesting that investment into one process results in decreased investment in the other. Prior work has demonstrated similar decreases in immunity during energetically taxing reproductive periods [11]–[13]. Sex effects detected in bacterial killing ability are also consistent with the immunocompetence hypothesis [58], which suggests that males exhibit decreased immune function relative to female conspecifics.

Between site effects suggest that an innate characteristic(s) of sites, such as the degree of urbanization, may contribute to altered physiology. We found a difference in magnitude of the change in CORT to stressor when comparing urban and rural populations, but no difference in baseline CORT among populations. It is important to note that our CORT data are measures of stress reactivity and while indicative of stress state does not specifically inform as to whether or not an individual is experiencing a chronic state of stress. More informative to the stress state of individuals are the related adverse effects of urbanization on immunity and survival; however, these effects might be offset by elevated reproductive output in urban populations. Furthermore, current results as to the effects of anthropogenic disturbances (i.e., urbanization, ecotourism) on stress reactivity in wild populations are mixed whereby some find elevated responses in human impacted areas relative to less disturbed populations [41], [59] while others find reduced responses in human impacted areas [52], [54], [60]. Similarly, some studies indicate that baseline CORT levels can be a strong predictor stress [45], and still other studies have demonstrated the opposite, indicating that the predictive value of baseline CORT may be species-specific, or possibly dependent on other factors, such as energetic state, life stage, and/or life history strategy [41, [61]. Given the previous variation in results we feel that measuring the comprehensive stress response in free-living organisms is most informative.

Our results reveal differences in bactericidal ability between populations located within St. George versus those located outside St. George. These results were consistent with other studies demonstrating altered immunity among urban populations [54]. Additionally, depressed immunity is associated with increased reproductive investment in urban lizards relative to their rural counterparts. Our wound healing results show similar effects in the relationship between immune function and urbanization. Healing injuries is known to be a stress-sensitive physiological response [52]–[64], and therefore suppressed healing coinciding with elevated stress responsiveness, such as those found in the urban populations we sampled, is not surprising and is consistent with prior studies [41].

### Mediation of Immune and Reproductive Tradeoffs by Stress

The provisioning of biological resources between competing physiological needs is a key function of the stress response [65]–[67]. For example, during emergency life history stages energetic investment is known to shift from reproduction to self-maintenance processes [21]. It is likely that elevated CORT during regular function, such as peak reproductive investment, serves to mobilize resources in preparation for increased energetic demands, particularly for females. Indeed one of the best characterized functions of CORT is in the mobilization of energy stores via processes such as gluconeogenesis [21]. Further, female fat stores become increasingly depleted during reproductive investment corresponding with deposition of deutoplasm into developing follicles [68], which also coincides with elevations in circulating CORT [69]–[70]. Due to the energetic debt experienced during reproductive investment, less energy is available for immunity, likely leading to a decrease in immune function. CORT’s role in energy mobilization, and known alterations in CORT response during energetically costly reproductive periods make this hormone a likely candidate for facilitating the observed seasonal shifts in immune investment in this species.

One potential mechanism for CORT’s initiation of changes in energy investment is stress-induced changes in oxidative stress. A recent meta-analysis clearly demonstrated the link between administered GCs and oxidative stress [71]. Our data demonstrates a similar relationship in response to endogenous GC concentration in free-living animals. Because both the development of oxidative damage and the ability to clear reactive oxygen metabolites are important components to oxidative stress, an index of animal’s plasma oxidative status, measured as a combination of both ROMs and OXY, can be used as a biologically relevant indicator of stress-induced effects to assess whether an animal is experiencing oxidative stress [55], [72]. We show that oxidative stress exhibits a positive relationship with circulating CORT, most likely driven by stress-induced increases in ROMs. Sustained physiological stress can also lead to the overproduction of ROMs [73]. If an individual’s antioxidant capacity is insufficient, ROMs can exert direct deleterious effects on tissues, and ultimately survival [19]–[20], such as by shortening telomere length, consequently resulting in an increased rate of senescence [74]–[76]. Populations experiencing heightened physiological stress may exhibit elevations in ROM presence that exceed the ability to clear those metabolites, potentially leading to accelerated senescence and subsequently resulting in profound population-level changes, such as the altered survival observed in this study. However, at this time we cannot rule out downstream consequences of altered immune activity and reproductive output as factors contributing to the direction of resource allocation or the observed population-level phenomenon.

### Conclusions

Although the partitioning of limited resources between competing processes has yet to be fully elucidated, research has shown that alterations in stress hormone concentration may contribute to the allocation of resources between competing processes [65]–[66]. Due to the fast-paced nature of short-lived species like *U. stansburiana*, life history theory predicts that during times of increased stress and decreased energy availability, resources will be reallocated toward reproduction at the cost of self-maintenance. Our results support this hypothesis by showing that increased CORT reactivity coincides with both increased reproductive output and decreased immune function. However, the direction of investment between self-maintenance and reproduction may be different for longer lived species, as their life history strategies would differ from fast-paced species. Additional studies incorporating both comparative and experimental approaches are necessary to determine the direction of resource partitioning across life history strategies, and whether stress physiology causally drives the direction of energetic investment between these processes. Perhaps the most significant finding of this study is that individual physiological changes can impact population-level processes, such as survival. Finally, while the urban environment poses definite challenges for most organisms [75], it appears that at least the side-blotched lizard may be able to compensate physiologically. However, until further longer-term studies are completed, it is unclear whether these physiological adjustments to the urban environment come at an ultimate cost of lifetime or population-level fitness.

## Methods

### Field Sampling

We sampled sexually mature adult male and female *U. stansburiana* of the same age class from the St. George, Utah, area (37° 5′ 43″ N, 113° 34′ 41″ W). Because lizard activity and CORT levels exhibit daily fluctuations, we sampled all individuals between 08∶00–12∶00. After capture via noosing we collected baseline blood samples from the retro orbital sinus using a heparinized capillary tube within 3 minutes of capture. We then placed individuals in an opaque bag for 10 minutes, after which a second “post-stress” blood sample was collected in the same fashion. Prior work has demonstrated significant stress-induced CORT elevations within 10 minutes of restraint stress in related lizard species [77]. By collecting baseline and post-stress blood samples, we were able to determine CORT response to capture and restraint stress, attaining a functional measure of individual stress response. We stored blood samples on ice until further processing could take place upon conclusion of daily collection, at which time we separated the plasma from cells via centrifugation and stored at −20°C until assayed.

We toe-clipped and marked all individuals with a unique code using a non-toxic paint pen for identification purposes. Using both manual palpitation of the abdomen to assess the number and firmness of follicles/eggs, as well as via high resolution ultrasound using a Sonosite MicroMaxx ultrasound unit with an external linear probe (a technique previously validated in this species [78]), we assessed female reproductive state. We used reproductive coloration to determine male breeding condition. We also recorded individual body mass and snout-vent-length (SVL), defined as the length from the tip of an individual’s snout to its cloacal vent. Upon completion of all sampling and data collection, we returned individuals to their point of capture.

### Ethics Statement

All handling and procedures were approved by the Utah State University Institutional Animal Care and Use Committee, protocol #1449. All field sampling was conducted under permission of the Utah State Division of Wildlife Resources, permit #1COLL8382.

### Study Sites

In an effort to collect physiological data from individuals experiencing varying levels of stress, we sampled individuals from four locations in and around St. George, Utah, across three months (April, May and June) in 2010, corresponding with early and peak reproductive periods for this species. Two sites were comprised of patch habitats located within St. George, that were exposed daily to human traffic in the form of recreational activities (running, jogging, biking, school groups, etc…). A third site was located in a rural area north of St. George, and the fourth site was located in a rural area within Gunlock State Park, southwest of St. George. All sites were of a similar habitat structure and size: sandy and rocky riparian zones of the same Virgin River drainage system.

### Self-maintenance Measures

#### Bactericidal assay

Working under a sterile laminar flow hood, we performed the bactericidal assay on individual plasma following procedures outlined in [79] and [53] with modifications, for use on a 96-well microplate, a 1∶12 plasma dilution with CO_2_-independent media (Gibco, Grand Island, NY) plus 4 mM L-glutamine (Sigma-Aldrich), 10^5^ CPU (colony-producing unit) *E. coli* (EpowerTM Microorganisms #0483E7, ATCC 8739, MicroBioLogics, St. Cloud, MN), and performing background absorbance corrections for each plate. Bactericidal capacity is calculated as the mean number of colonies for each sample that were run in duplicate, divided by the mean of colonies for the positive controls (three plates containing only media and bacterial solution), and multiplied by 100 (i.e., % bacteria killed relative to the positive control). Bactericidal assays were performed on baseline plasma samples (inter-plate CV 2.74%).

#### Cutaneous wound biopsy and analysis

Using procedures outlined in [80], we administered a uniform cutaneous wound on the dorsal side of each individual using a biopsy punch, and digitally photographed wounds using a Pentax K-x digital camera. Animals were recaptured and re-photographed 10 days after biopsy following [80]. At the time of recapture, we also collected baseline and post-stress blood samples. All blood samples were processed for RIA and bactericidal assay per the procedures described above. Images were randomized and analyzed to assess wound size (i.e., area) at the start and end of the study using image analysis software (Image J, NIH Imaging), such that the investigator was blind to the treatment of the animal.

#### Oxidative Stress

To measure ROMs we used a kit (Diacron, Grosseto, Italy) that detects the level of hydroperoxides, ROMs that signal lipid and protein oxidative damage [81], [82]. We diluted 5 µl of plasma into 100 µl of the provided acidic buffered solution and followed the ‘end-point mode’ manufacturer instructions, with modifications for use on 96-well microplate. Inter-plate variation was 1.68%, and intra-plate variation was 0.30% on duplicate samples.

Antioxidant capacity was measured using the OXY-Adsorbent test (Diacron, Grosseto, Italy), which measures the effectiveness of the blood antioxidant barrier by quantifying its ability to cope with the oxidant action of hypochlorous acid (HClO) [55], [72]. Briefly, we diluted 2 µl of plasma in 100 µl of distilled water and followed manufacturer instructions with modifications for use on a microplate. We then mixed a 5 µl subsample of this diluted plasma with 100 µl of the HClO solution provided. Inter- and intra-plate variations were ∼1.35% and 0.70%, respectively.

### Reproductive Measures

Because our preliminary validation demonstrated no statistically significant differences in estimates of egg/follicle size using ultrasound imaging compared to measurements taken during laparotomy (*P*>0.05), egg/follicle size was estimated using the length and diameter of the largest egg detected on the ultrasound, a measure known to be directly related to egg volume.

### Radioimmunoassay

We quantified circulating hormone levels using a previously described and established radioimmunoassay (RIA) protocol [83] with a slight modification; extractions were performed using a solution of 30% ethyl acetate : isooctane. Following extraction, using the same protocols as Moore [83], we dried the samples, resuspended them in PBS buffer, and assayed in duplicate for CORT (Fitzgerald 20-CR45, Lot #P0012502). For each sample we used an aliquot of the resuspended fractions to measure individual recoveries following extraction and chromatography. These recoveries are used to adjust the final sample concentration values to account for any losses during these procedures. Five separate assays were performed for all of our samples, with an inter-assay variation of 14.3%, and mean intra-assay variation of 10.4%.

### Survival Estimates

We estimated adult survival using a robust design Capture-Mark-Recapture (CMR) technique [84] and Program MARK 6.1 software. As previously described, we toe-clipped and marked 233 individuals from 4 study sites (Urban 1 *N* = 56; Urban 2 *N* = 85; Rural 1 *N* = 28; Rural 2 *N* = 64). We recorded the initial capture, and tracked each individual for resightings over 8 sampling occasions in April, May and June 2010. Due to logistical limitations, blood samples were not collected for each individual, thus a population mean for each CORT and bactericidal ability was calculated and used for each individual in the analyses.

The Akaike Information Criterion corrected for small sample sizes (AIC_c_) was used for model selection [85]. Because AIC_c_ uses a balance between precision in parameter estimation and model fit, lower AIC_c_ values indicate models best supported by the data [86]. The differences between models (ΔAIC_c_) is considered to be statistically significant if the difference is greater than 2, while a ΔAIC_c_ of less than 2 assumes that models are similar [86]. Akaike weights (w_i_) were used as an index of the relative support for each model. Survival estimates *φ(x)* were calculated by back-transforming beta estimates *β(x)* generated from candidate models on a logit scale (i.e. the logit function transforms beta estimates into survival probabilities constrained between 0 and 1), such as: 
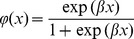
. We also provided 95% confidence intervals for both beta estimates and real survival estimates to assess the significance of covariates (i.e., CORT and bactericidal ability) on survival (i.e., effects were significant if 95% confidence intervals did not overlap 0, and vice versa).

### Statistical Analyses

Unless otherwise stated, the significance level for all statistical tests was α = 0.05, and all data are presented as mean ± standard error (SE). Statistical analyses were conducted using JMP.IN (v. 8.0.1, SAS Institute Inc., Cary, NC, USA), which automatically accounts for unequal sample sizes. Hormonal data were log-transformed, while both bactericidal and wound healing data were arcsin-squareroot transformed to satisfy the assumptions of normality. All pairwise comparisons were detected using Student’s *t* post-hoc comparisons.

Before performing more complex statistical analyses, we first determined whether there were differences in body condition across sites. Individual body mass was regressed against SVL to generate residuals equivalent to “body condition.” One-way Analysis of Variance (ANOVA) failed to detect any differences in body condition across sites (*F_3,171_* = 0.15, P>0.05), suggesting that statistical differences detected across sites during additional analyses were not simply an artifact of site-level differences in body condition.

## Supporting Information

Table S1 Candidate model analysis summaries. Nine candidate models were tested to estimate survival probability with Program MARK. The best model tested included CORT reactivity as a covariate. In addition to CORT reactivity, the second and third best models also included bactericidal ability and individual sex as covariates, respectively. Although both of these models are within 2 ΔAIC_c_ of the best model, the AIC_c_ weight of the best model indicates it supported approximately 2–2.5 times as much by the data as these subsequent models. Further, because the confidence intervals of the beta parameters of these candidate models overlap zero, these models are most likely not statistically significant and survival estimates from these models may not be accurate. If the second and third best models are discarded for these reasons, the next best model is one which only includes bacterial killing ability as a covariate. The beta parameters of this model does not overlap zero, suggesting statistical significance. However, given that the ΔAIC_c_ between this model and the best model is greater than 2, the difference between these models is statistically significant. However, it should be noted that the survival estimates of the candidate model including bacterial killing ability as a covariate shows similar trends as those of the best model (Table 1). Thus, though our data suggests that CORT reactivity exerts the greatest influence on survival, bactericidal ability may still be a significant factor. The inaccurate estimates provided by the model that includes both CORT reactivity and bacterial killing ability may actually be an artifact of not having enough power to include two covariates into our model. Because the model which only includes individual sex as a covariate performed worse than the null model, it would seem that sex most likely is not a significant factor contributing to survival. At least one beta parameter of the covariates for all other models overlapped zero, suggesting that statistical significance for those models are unlikely. Further, given the ΔAIC_c_ of the best model and all other models, and a comparison of their AIC_c_ weights, our data strongly supports the model that includes only CORT reactivity as a covariate, suggesting that CORT response is an important driving factor in the variation in survival observed in our populations of *U. stansburiana*.
(DOCX)
