# Supplementary material for: Stress-Induced Tradeoffs in a Free-Living Lizard across a Variable Landscape: Consequences for Individuals and Populations
Source: PLoS One. 2012 Nov 20;7(11):e49895. doi: 10.1371/journal.pone.0049895 (PMC3502225; doi:10.1371/journal.pone.0049895)
Supplement: Table S1 — Candidate model analysis summaries. Nine candidate models were tested to estimate survival probability with Program MARK. The best model tested included CORT reactivity as a covariate. In addition to CORT reactivity, the second and third best models also included bactericidal ability and individual sex as covariates, respectively. Although both of these models are within 2 ΔAICc of the best model, the AICc weight of the best model indicates it supported approximately 2–2.5 times as much by the data as these subsequent models. Further, because the confidence intervals of the beta parameters of these candidate models overlap zero, these models are most likely not statistically significant and survival estimates from these models may not be accurate. If the second and third best models are discarded for these reasons, the next best model is one which only includes bacterial killing ability as a covariate. The beta parameters of this model does not overlap zero, suggesting statistical significance. However, given that the ΔAICc between this model and the best model is greater than 2, the difference between these models is statistically significant. However, it should be noted that the survival estimates of the candidate model including bacterial killing ability as a covariate shows similar trends as those of the best model (Table 1). Thus, though our data suggests that CORT reactivity exerts the greatest influence on survival, bactericidal ability may still be a significant factor. The inaccurate estimates provided by the model that includes both CORT reactivity and bacterial killing ability may actually be an artifact of not having enough power to include two covariates into our model. Because the model which only includes individual sex as a covariate performed worse than the null model, it would seem that sex most likely is not a significant factor contributing to survival. At least one beta parameter of the covariates for all other models overlapped zero, suggesting [file pone.0049895.s001.docx]

| Model | AIC_c_ | ΔAIC_c_ | AIC_c_ Weight | Parameters | -2Log(L) |
| --- | --- | --- | --- | --- | --- |
| φ(CORT) | -76.1960 | 0.0000 | 0.35959 | 11 | -98.9038 |
| φ(CORT+BKA) | -74.6189 | 1.5771 | 0.16344 | 12 | -99.4576 |
| φ(CORT+Sex) | -74.4263 | 1.7697 | 0.14843 | 12 | -99.2650 |
| φ(BKA) | -73.7099 | 2.4861 | 0.10374 | 11 | -96.4177 |
| φ(CORT+BKA+Sex) | -72.7938 | 3.4022 | 0.06562 | 13 | -99.7749 |
| φ(CORT+BKA+CORT*BKA) | -72.5731 | 3.6229 | 0.05876 | 13 | -99.5543 |
| φ(BKA+Sex) | -72.0338 | 4.1622 | 0.04487 | 12 | -96.8725 |
| φ(.) | -71.7251 | 4.4709 | 0.03846 | 10 | -92.3134 |
| φ(Sex) | -70.1016 | 6.0944 | 0.01708 | 11 | -92.8094 |
